# Evaluation of Growth and Health Performance of Juvenile Tilapia *Oreochromis* sp. Fed with Various Supplementation of Heat-Killed *Lactobacillus plantarum*

**DOI:** 10.1155/2023/8860364

**Published:** 2023-08-22

**Authors:** Julie Ekasari, Vini Nur Mirza, Ichsan Achmad Fauzi, Kyohei Hashimoto, Muhammad Agus Suprayudi

**Affiliations:** ^1^Department of Aquaculture, Faculty of Fisheries and Marine Sciences, IPB University, Bogor, Indonesia; ^2^House Wellness Foods Corporation, Hyogo, Japan

## Abstract

Thus, this research was conducted to evaluate the supplementation of heat-killed *Lactobacillus plantarum* at higher dosages and investigate the effect of heat-killed *L. plantarum* supplementation on the challenges of *Streptococcus agalactiae*. A feeding trial was conducted for 90 (initial average body weight of 12.52–12.69 g) days, while a disease challenge was conducted for 17 days. Dietary treatments were formulated to have a ranging level of heat-killed *L. plantarum* L-137: (1) control treatment without heat-killed *L. plantarum*, (2) diet containing 10 mg/kg heat-killed *L. plantarum* L-137 preparation (LP20, which contains 20% heat-killed *L. plantarum* L-137), (3) diet containing 20 mg/kg LP20, (4) diet containing 100 mg/kg LP20, and (5) diet containing 250 mg/kg LP20. All the diets were formulated to have equal values of protein and energy. Dietary supplementation of heat-killed *L. plantarum* L-137 improved tilapia growth performance and higher robustness against *S*. *agalactiae* infection. Therefore, a 10–20 mg/kg LP20 feed supplementation level is recommended to support the tilapia growth. In addition, an LP20 dietary supplementation level of 250 mg/kg feed is recommended for higher protection against *S. agalactiae*.

## 1. Introduction

Within the next decade, aquaculture will be more critical in fulfilling the increasing demand for fish and aquatic commodities, especially since the capture fisheries sector stagnates due to its maximum production yield [1]. Aquaculture commodities like tilapia (*Oreochromis niloticus*) are considered essential aquaculture products that can provide high protein for human consumption at a relatively lower price. Based on data from The State of World Fisheries and Aquaculture [1], tilapia is considered one of the top three finfish commodities, with a total production in 2020 of more than 4.4 million tons and with a percentage share of around 9% of the total finfish production in the world. This commodity is also considered one of the most versatile fish. It can live in various environments and is considered one of the more sustainable aquaculture commodities [2].

One obstacle that hinders the global production of tilapia is bacterial diseases such as streptococcosis caused by *Streptococcus agalactiae* considered the most common bacterial disease that causes significant loss to the tilapia industry [3]. A report from the study by Sun et al. [4] stated that *S. agalactiae* infection caused high-cumulative mortality in tilapia farms, consequently leading to the economic loss. Furthermore, streptococcosis commonly presents in intensive tilapia culture systems since the prevalence of this disease will increase in the case of low-dissolved oxygen and high-ammonia level [3]. Therefore, one solution that can be conducted to overcome the streptococcosis problem in tilapia is using functional ingredients that can increase the health status of the fish.

One functional ingredient that has been proven beneficial for various fish performance when supplementing through the feed is probiotics. The application of probiotics in the aquaculture field has been used and evaluated extensively. The effect of probiotics on growth performance has been evaluated by the multiple researchers [5–10]. Of many potential species candidates for probiotics, *Lactobacillus plantarum* had already demonstrated positive effects in the studies conducted on various fish. For example, a study was conducted on rohu fish (*Labeo rohita*) [11], orange-spotted grouper (*Epinephelus coioides*) [12], and Nile tilapia (*Oreochromis niloticus*) [13] had shown improvement on growth and immune parameters when *L. plantarum* mixture was added to the diet at a different dosage. However, special considerations are required when adding live bacteria into the commercial pelleted diet since exposure to high temperatures, primarily when the diet is produced with the extrusion process, may kill probiotic bacteria due to the heat [14].

Another use of *L. plantarum* is through a heat-treated method to form paraprobiotic. Paraprobiotic is the term proposed to describe using cell components of probiotics by using inactivated probiotics that can confer health benefits to the host [15]. The use of *L. plantarum* paraprobiotic has been evaluated before in several species such as snakehead fish, *Channa striata* [16], amberjack, *Seriola dumerili* [17], red sea bream, *Pagrus major* [18], and kuruma shrimp, *Marsupenaeus japonicus* [19, 20]. In the case of Nile tilapia, previous research reported has already reported the efficacy of heat-killed *L. plantarum* on the growth performance and immune status at 10 and 20 mg/kg, respectively [21]. However, the efficacy of heat-killed *L. plantarum* at lower than 10 mg/kg are not evaluated yet. Thus, this research was conducted to evaluate the supplementation of heat-killed *L. plantarum* L-137 at a lower supplementation dosage and investigate the effect of heat-killed *L. plantarum* L-137 supplementation on the challenges of *S. agalactiae*.

## 2. Materials and Methods

### 2.1. Rearing Condition

This study was conducted in two phases: a feeding trial for 90 days and a disease challenge for 17 days using *S. agalactiae* at the end of the feeding trial. Juvenile tilapia in this trial were acquired from the Aquaculture Research and Teaching Facility, Department of Aquaculture, IPB University. The experimental procedures in this study have complied with the ethical guidelines from the Animal Care and Use Committee of IPB University, Indonesia. Tilapia was first acclimated for 14 days in the rearing facility. During this period, the juvenile tilapia were fed using commercial diets with 28.1% and 7.2% crude protein and fat content.

Meanwhile, the diet used during the feeding trial was a formulated diet that included intact protein such as fish meal, poultry byproduct meal, meat bone meal, and soybean meal. The diet's dietary formulation and nutrient analysis can be seen in Table 1. The average size of the tilapia at initial stocking was 12.61 with a standard deviation of ± 0.18 g, distributed randomly to each tank at a density of 20 fish per tank. Tilapia was first acclimated for 14 days in the rearing facility. At this period, the juvenile tilapia were fed using commercial diets Hi-PRO-Vite 788 (CP Prima, Jakarta, Indonesia) with 28.1% crude protein content and 7.2% crude fat content. The tanks used in this research were rectangular glass aquariums with a total working volume capacity of 200 L. All the aquariums were equipped with a top filter to remove organic particles from the water and a thermostat to maintain water temperature for optimum growth. During the feeding trial, water quality was maintained by water exchanges once every 3 days or when necessary. Water temperature, pH, and dissolved oxygen (DO) were monitored daily in the morning before and in the afternoon before feeding using a thermometer, portable pH meter, and DO meter, respectively. Nitrite concentrations were measured once every 3 days, while total ammonia nitrogen (TAN) and nitrate were measured every two weeks. Measurement of TAN, nitrite, and nitrate was conducted in accordance with Eaton et al. [23]. All the water quality measurements can be found in the Supplementary Table S1.

### 2.2. Dietary Treatment and Feeding Management

Dietary treatments were formulated to have a ranging level of LP20 (House Wellness Food Corporation, Hyogo, Japan) which contains 20% heat-killed *L. plantarum* L-137: (1) control treatment without LP20, (2) diet containing 10 mg/kg heat-killed *L. plantarum* L-137 which contain 2 mg/kg heat-killed *L. plantarum*, (3) diet containing 20 mg/kg LP20 which contain 4 mg/kg *L. plantarum*, (4) diet containing 100 mg/kg LP20 which contain 20 mg/kg *L. plantarum*, and (5) diet containing 250 mg/kg LP20 which contain 50 mg/kg *L. plantarum*. Each treatment was conducted in four replicates of tanks. All the diets were formulated to have an equal value of protein and energy and consist of an intact protein source such as fish meal, poultry byproduct meal, meat and bone meal, and soybean meal. The complete formulation of all the diets can be seen in Table 1. Diets were developed at Aquaculture Research and Teaching Facility's Feed mill, IPB University. Diets were developed by mixing all the ingredients while adding water using an industrial mixer until the dough was homogenous and could be shaped into a pelleted form (diameter of 3 mm) using a pellet machine. Afterward, the pellet was dried overnight using an oven with a temperature of 50°C. The diets were then stored in a cool and dry place using the plastic containers. The nutritional value of the diets was then confirmed through proximate analysis according to methods by AOAC Int [24]. During this trial, feeding was conducted three times a day. The feed was given to each tank until the fish's apparent satiation. Feeding was slowed down when feeding responses from the fish were declining to ensure all the feed was eaten by the fish and avoid an accumulation of uneaten feed at the bottom of the tanks.

### 2.3. Sampling

The fish's biomass and survival were monitored every 2 weeks by weighing all the fish and counting the number of fish left in every tank. The amount of feed given to each aquarium was also recorded to evaluate the total feed intake and feed conversion rate. At the end of the trial, intestines were also collected from one fish per tank and transferred into a neutral buffered formalin before being processed for histological analysis.

The average daily weight gain, feed conversion ratio, and fish survival were calculated given below formula: average daily weight gain (DWG): (final average fish weight-initial average fish weight)/days of the feeding trial, feed conversion ratio (FCR): total weight of feed given (kg)/(final biomass (kg) + biomass of dead fish−initial biomass), survival: the number of the fish at the end of feeding trial/the number of the fish at initial feeding trial × 100%.

### 2.4. Observed Parameters

#### 2.4.1. Blood Collection and Challenge Test

A challenge test using *S. agalactiae* was performed after the growth experiment followed by another hematological parameter observation. The measurement of hematological parameters was conducted following the procedures described by Suprayudi et al. [25]. Blood samples were taken from the caudal arch of anesthetized fish (150 mg/L tricaine methane sulfate) using a 25-gauge needle and a 3 mL heparinized syringe. Blood analyses were done following Blaxhall and Daisley [26].

Before the challenge test, a preliminary experiment was conducted to determine the LD50 (1 × 10^6^ CFU/mL) of *S. agalactiae*. For the challenge test, ten healthy fish were selected from each replicate tank and transferred into another tank. The fish were anesthetized and intramuscularly injected with 1 mL of the bacteria suspension (1 × 10^6^ CFU/mL). As a negative control, 10 healthy fish were collected from the control treatment and injected with 1 mL of PBS. The challenge test was performed for 17 days, and blood samples were collected to measure the blood parameters.

#### 2.4.2. Intestine Histology

After 90 days of fish rearing and 14 hr of fasting, the proximal part of the intestines was collected and transferred to a 10% neutral buffered formalin. The histological analysis was conducted using the hematoxylin–eosin staining method, and morphometrics of the intestine was observed under a microscope to measure the mucosal fold height and mucosal fold's surface area. Mucosal fold measurements were conducted using ImageJ software (National Institute of Health, Bethesda, USA).

#### 2.4.3. Statistical Analysis

Fish growth performance parameters, intestinal histomorphometry, and hematological parameters were analyzed using one-way analyses of variance (ANOVA) followed by the Duncan post hoc test. Data normality and homogeneity were assessed before ANOVA analysis using the Shapiro–Wilk normality test and Bartlett test. The survival data were arcsine transformed, and postchallenged survival data were analyzed using Kaplan–Meier survival analysis. Statistical analyses were performed using GraphPad Prism (GraphPad Software, San Diego, USA).

## 3. Results

### 3.1. Growth Performance

**Figure 1:**
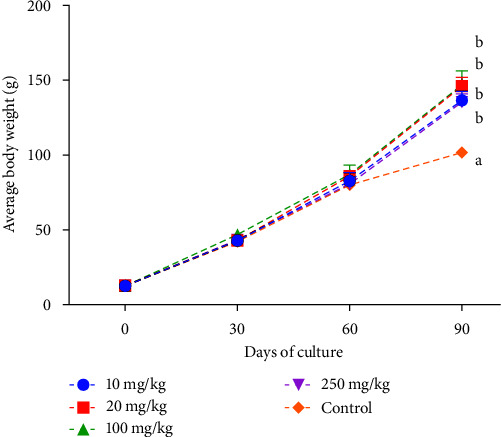
Growth of tilapia fed with different dosage of LP20. Different letter in the graph indicates significant differences among treatment. Error bar in the graph demonstrate standard error of the mean (SEM). Some of the error bars were too small so that it cannot be shown in the graph.

Growth performance parameters (average daily gain, specific growth rate, final total length, and length gain) indicate a significantly higher value in treatments supplemented with LP20 than in control treatment (Table 2) and significant differences were only found at 90 days sampling period (Figure 1). No significant differences were found in the case of survival among all treatments.

While the control group demonstrated a higher feed conversion ratio and lower protein efficiency ratio, no significant differences were found among all treatments in the case of feed and protein utilization. No significant differences were found among treatments on the protein and lipid retention parameters, however, feed intake parameters in both total fish intake and average feed intake demonstrate significantly higher value on treatments with supplementations of LP20 compared to the control treatment (Table 2).

In the case of fish biochemical indices (Table 3), significant differences were found in the whole-body moisture and crude protein content. In whole-body moisture, the lowest value was found in the treatment with 20 mg/kg LP20, and it was significantly different from the control treatment, with 10 and 100 mg/kg LP20. In the case of protein, higher protein values were found in the treatment with 20 mg/kg LP20, which was significantly different compared to 10 mg/kg LP20 and the control treatment.

In the mucosal fold histomorphology (Table 4), significant differences in height were observed after 90 days of sampling. In contrast, there were significant differences between treatments with 10 and 250 mg/kg LP20, although no differences were found in other treatments.

**Figure 2:**
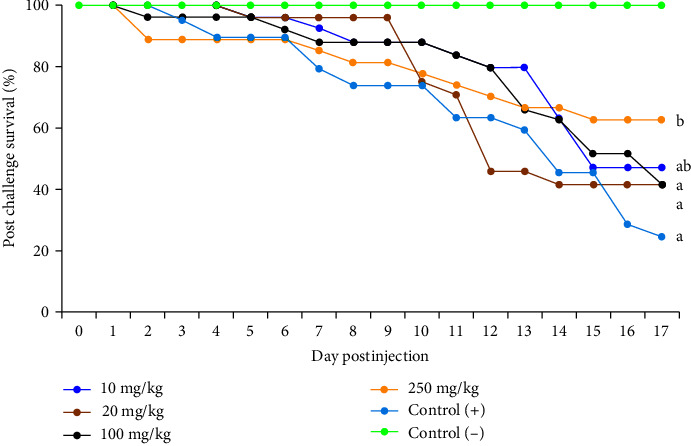
Survival of tilapia fed with diets supplemented with different levels of LP20 upon challenged *Streptococcus agalactiae*; different superscript letters indicate significant differences at *P* < 0.05.

At 7 days postchallenge (Table 5), a higher than positive control group was found in the white blood count of treatment with 100 and 250 mg/kg LP20 treatment, while a lower value of the red blood cell was found in the tilapia fed with 10 and 20 mg/kg LP20 compared only to the negative control. Moreover, in the postchallenged survival, a statistically higher value was found on treatment with 250 mg/kg LP20 compared to treatment with 0 (control), 20, and 100 mg/kg of LP20 at 17 days postinjection (Figure 2).

## 4. Discussion

Compared to the study by Van Nguyen et al. [21], this research demonstrated that lower supplementation of LP20 (at least 10 mg/kg) could increase the growth performance of juvenile tilapia. However, higher supplementation up to 250 mg/kg did not improve growth performance compared to 10 mg/kg. This finding is also in line with research conducted by Hien et al. [27] on bighead catfish (*Clarias macrocephalus*), which demonstrated a higher specific growth rate when fed with dietary supplementation of 10 mg/kg LP20 and no further performance increase when supplementation levels are increased up until 50 mg/kg of LP20. While there are differences in growth performance, there are no differences in the feed conversion ratio among all the treatments. The effect of dietary inclusion of heat-killed *L. plantarum* is varied since other studies found confounding results on the feed conversion rate when this paraprobiotic was added. In the research conducted by Dawood et al. [17] in amberjack (*Seriola dumerili*) and Van Nguyen et al. [21] in tilapia, the dietary addition of heat-killed *L. plantarum* L-137 did not affect the feed conversion rate. Meanwhile, other studies on snakehead, *Channa striata* [16], red sea bream, and *Pagrus major* [17] demonstrate better conversion rates when heat-killed *L. plantarum* L-137 is added to the diet.

Better growth performance in treatments on supplementation of heat-killed *L. plantarum* and a similar level of feed conversion ratio among all the treatments in this research might be related to a significant difference in feed intake of treatments with supplementation of heat-killed *L. plantarum*. Therefore, it is possible that supplementation of the ingredients can affect the palatability of the diet thus improving feed intake especially since the diet that was formulated in this trial was using a low-fish meal and high plant-based ingredients. A higher feed intake in the case of supplementation dietary with heat-killed *L. plantarum* was also found in other studies, in the study conducted by Van Nguyen et al. [21] and Hien et al. [27] demonstrate a higher feed intake when tilapia was fed with 4 mg/kg of heat-killed and 20 mg/kg of heat *L. plantarum*, respectively.

One possible quantitative parameter that can measure changes in the intestinal morphometric is mucosal fold length and mucosal fold surface area. Previous research has concluded that the increased gut morphometric value in the fish fed with supplementation of heat-killed *L. plantarum* L-137 is related to increased absorptivity and can consequently result in increased nutrient utilization [28]. In this research, although the average value of mucosal folds surface area on treatment heat-killed *L. plantarum* was higher, significant differences were only found in treatment with 20 mg/kg of LP20 compared to the control treatment. Thus, it is possible that the difference in mucosal fold histometric is one of the factors that contribute to better growth performance in this study. Aside from changes in mucosal fold histometric, the dietary inclusion of heat-killed *L. plantarum* can also affect the diversity of gut microbiota, as shown in research by Wu et al. [29]. While the changes in gut microbes were not evaluated in this study, it is possible that gut microbiota composition also contributes to the growth difference among those treatments.

Our research demonstrated that after being challenged with *S. agalactiae*, the leucocytes of treatments with 100 and 250 mg/kg LP20 were significantly higher than the positive control. The higher level of leucocyte, along with better survival upon challenge with *S. agalactiae*, demonstrates the capability of LP20 to improve immune function when supplemented at a higher dosage. Previous research on snakehead fish demonstrated higher immunocompetence and resistance to *Aeromonas hydrophila* when the fish was fed with dietary supplementation of heat-killed *L. plantarum* L-137 [16]. Interestingly, in the same research, a lower dosage of heat-killed *L. plantarum* L-137 at 2 mg/kg (10 mg/kg LP20) is enough to improve the immune performance of snakeheads. However, whether the dosage difference found in the snakehead and tilapia was due to differences in rearing conditions or related to different species is still unclear.

Upon being challenged with *S. agalactiae*, survival analysis indicates better resistance on treatment that was fed with 250 mg/kg LP20 compared to the positive control. The beneficial effect of dietary heat-killed *L. plantarum* L-137 is in line with other research conducted on tilapia [21], red sea bream [18], and snakehead [16]. While research on various species demonstrates improvement in resistance upon challenge test, the optimum dosages for immune and disease resistance differed from previous research. In the study conducted on the sea bream [18], a dosage of 10 mg/kg of LP20 is enough to demonstrate a longer time to reach 50% cumulative mortality. Thus, increasing the dosage up to 1,000 mg/kg further improves the time required to achieve 50% cumulative mortality. In the research conducted on snakehead [16], fish that was fed with 2 mg/kg heat-killed *L. plantarum* L-137 (10 mg/kg LP20) demonstrated better performance compared to the other treatments, meanwhile increasing the dietary dosage of heat-killed *L. plantarum* L-137only increase the cumulative mortality even though the number is still significantly lower compared to positive control that was fed with control diet without the addition of heat-killed *L. plantarum* L-137. Furthermore, in the previous research on tilapia [21], better resistance to *S. agalaticea*e can be found only at treatment with dietary supplementation of heat-killed *L. plantarum* L-137 at 20 mg/kg, increasing the dosage until 50 mg/kg did not produce better survival compared to the treatment diet. The highest dosage that they use is 50 mg/kg of heat-killed *L. plantarum* L-137 while our research indicates that supplementation at around 250 mg/kg LP20 (50 mg/kg heat-killed *L. plantarum* L-137) can improve tilapia's survivability upon challenged with *S. agalactiae*. It is possible, the different dosages that can improve tilapia's survivability can be affected by different rearing systems, Van Nguyen et al. [21] study was using a flow-through system while ours only used a top filter with occasional water exchange periodically, thus the water quality especially in the case of ammonia and nitrite in the rearing system may affect efficacy of the para-probiotic product. Therefore, the effect of water quality on the efficacy of heat-killed *L. plantarum* L-137 should be further elaborated in another study.

While this research has shown that the optimum dosage of heat-killed *L. plantarum* L-137 can be varied according to the effect on the growth performance or enhancement of the immune system, supplementation of inactive *L. plantarum* L-137 in the form of paraprobiotic can also be as efficient as supplementation of *L. plantarum* L-137 in the form of probiotic. Research in the utilization of *L. plantarum* as a probiotic by Giri et al. [11] on the rohu (*Labeo rohita*) and Son et al. [12] on the orange-spotted grouper (*Epinephelus coioides*) demonstrate similar growth and immune performance compared to the use of heat-killed *L. plantarum*. Furthermore, heat-killed *L. plantarum* is more straightforward to store and handle than the live probiotic form.

In conclusion, Dietary supplementation of heat-killed *L. plantarum* L-137 resulted in improvements in tilapia growth performance and higher robustness against *S. agalactiae* infection. Therefore, a supplementation level of 10–20 mg/kg LP20 feed is recommended to support the growth of tilapia. In addition, an LP20 dietary supplementation level of 250 mg/kg feed is recommended for higher protection against *S. agalactiae*.

## Figures and Tables

**Table 1:** Dietary formulation and nutrient composition of the treatment diet.

	10 mg/kg	20 mg/kg	100 mg/kg	250 mg/kg	0 (control)
Ingredients (crude protein/crude lipid content on dry base) g/kg^†^					
Fish meal (58.8/11.9)	50	50	50	50	50
Poultry byproduct meal (50.0/12.9)	45	45	45	45	45
Meat bone meal (51.8/9.8)	65	65	65	65	65
Soybean meal (48.2/1.6)	260	260	260	260	260
Wheat flour (11.9/3.5)	135	135	135	135	135
Wheat pollard (15.9/4.5)	280	280	280	279.8	280
Cassava flour (3.1/0.8)	40	40	40	40	40
Copra meal (21.5/16.5)	50	50	50	50	50
Fish oil	15	15	15	15	15
Crude palm oil	20	20	20	20	20
Vitamin mix	10	10	10	10	10
Mineral mix	30	30	30	30	30
LP20^‡^	0.01	0.02	0.1	0.25	0
Nutrient composition (% dry weight based)					
Moisture (%)	13.32	14.15	15.46	12.19	9.87
Crude ash (%)	10.51	11.63	12.83	10.54	11.03
Crude protein (%)	29.41	29.68	29.63	31.53	28.19
Crude fat (%)	5.02	6.42	5.39	6.81	7.22
Crude fiber (%)	5.03	5.28	4.29	3.53	5.23
Non-nitrogen extract (%)	36.71	32.85	31.4	35.4	38.46
Gross energy (Kcal/kg)	3591.80	3573.06	3414.70	3815.94	3801.82

*Note*: ^†^All the ingredients except for LP20 were provided from PT Wonokoyo Jaya Kusuma, Serang, Indonesia. ^‡^Vitamin premix composition (Suprayudi et al. 2014): retinol (A), 900 IU kg; ascorbic acid (C), 200 mg kg^−1^; cholecalciferol (D), 200 IU kg^−1^; menadione (K3), 10.0 mg kg^−1^; a-tocopherol (E), 100 mg kg^−1^; choline, 1,000 mg kg^−1^; inositol, 100 mg kg^−1^; thiamin (B1), 15 mg kg^−1^; riboflavin (B2), 20 mg kg^−1^; pyridoxine (B6), 15 mg kg^−1^; d-pantothenic acid (B5), 50 mg kg^−1^; nicotinic acid, 75 mg kg^−1^; biotin, 0.5 mg kg^−1^; cyanocobalamin (B12), 0.05 mg kg^−1^; folic acid, 5 mg kg^−1^; Mineral premix composition (Suprayudi et al. 2014): Co (asCoCl2_6H2O), 0.5 mg kg^−1^; Cu (as CuSO4_5H2O), 5 mg kg^−1^; Fe (as FeSO4_7H2O), 50 mg kg^−1^; I (as KI), 4 mg kg^−1^; Cr (as CrCl3_6H2O), 0.1 mg kg^−1^; Mg (as MgSO4_7H2O), 150 mg g^−1^; Mn (as MnSO4_H2O), 25 mg kg^−1^; Se (as NaSeO3), 0.1 mg kg^−1^; Zn (as ZnSO4_7H2O), 100 mg kg^−1^. Amino acid mix, 0.3 g kg^−1^; Natrium chloride (NaCL), 1 g kg^−1^. LP20: Preparation containing 20% heat-killed *L. plantarum* L-137 (House Wellness Foods Corporation, Hyogo, Japan). ^§^Gross energy were calculated in accordance with [22]. 1 g protein: 5.6 Kcal Kg^−1^ GE, 1 g lipid: 9.4 Kcal Kg^−1^ GE, 1 g of non-nitrogen extract: 4.2 Kcal Kg^−1^ GE.

**Table 2:** Growth parameters, total feed intake, survival, feed conversion ratio, protein retention, and lipid retention (mean ± SE) of red tilapia fed with supplementation of LP20.

	10 mg/kg	20 mg/kg	100 mg/kg	250 mg/kg	0 (control)
IW (g)	12.52 ± 0.10	12.67 ± 0.23	12.58 ± 0.20	12.69 ± 0.24	12.61 ± 0.13
FW (g)	137 ± 13^a^	147 ± 10^a^	147 ± 19^a^	135 ± 11^a^	102 ± 5^b^
B_t_ (kg)	1.60 ± 0.16	1.65 ± 0.10	1.65 ± 0.23	1.53 ± 0.20	1.27 ± 0.12
DWG (g/day)	1.36 ± 0.13^a^	1.47 ± 0.11^a^	1.48 ± 0.21^a^	1.35 ± 0.12^a^	0.98 ± 0.05^b^
SGR (%/day)	2.62 ± 0.10^a^	2.68 ± 0.09^a^	2.69 ± 0.15^a^	2.60 ± 0.09^a^	2.29 ± 0.06^b^
TFI (kg)/AFI (g/fish)	2.26 ± 0.18^a^/192.3 ± 11.7^a^	2.34 ± 0.06^a^/207.0 ± 12.8^a^	2.34 ± 0.19^a^/208.0 ± 15.7^a^	2.19 ± 0.10^ab^/195.3 ± 11.5^a^	1.92 ± 0.09^b^/154.2 ± 6.6^b^
FCR	1.36 ± 0.06	1.32 ± 0.08	1.31 ± 0.09	1.34 ± 0.08	1.40 ± 0.06
PER	2.13 ± 0.08	2.13 ± 0.16	2.12 ± 0.19	2.07 ± 0.22	1.91 ± 0.15
TL (cm)	19.33 ± 0.62^a^	19.68 ± 0.61^a^	19.71 ± 0.89^a^	19.08 ± 0.61^a^	17.35 ± 0.19^b^
LG (cm)	9.86 ± 0.62^a^	10.21 ± 0.61^a^	10.24 ± 0.89^a^	9.61 ± 0.61^a^	7.88 ± 0.19^b^
PR (%)	40.15 ± 0.81	44.70 ± 2.21	40.33 ± 1.94	38.40 ± 2.08	40.50 ± 0.81
LR (%)	70.68 ± 4.24	68.63 ± 1.62	66.21 ± 8.84	58.81 ± 4.40	51.04 ± 0.88
Survival (%)	98.33 ± 3.33	95.56 ± 3.85	95.00 ± 3.33	95.00 ± 6.38	88.33 ± 3.33

*Note*: IW, initial body weight; FW, final body weight; B_t_, final biomass; BDWG, average daily weight gain; SGR, specific growth rate; TFI, total feed intake; AFI, average feed intake; FCR, feed conversion ratio; PER, protien efficiency ratio; TL, total length; LG, length gain; PR, protein retention; LR, lipid retention. The values presented were average ± standard error (*n* = 4). Different superscript letters indicate significant differences at *P* < 0.05.

**Table 3:** Whole body moisture, crude protein, crude lipid, and ash content of tilapia fed with different dietary supplementation of LP20.

Composition (% wet based)	10 mg/kg	20 mg/kg	100 mg/kg	250 mg/kg	0 (control)
Moisture	73.02 ± 0.29^a^	70.22 ± 0.39^b^	73.04 ± 0.74^a^	71.91 ± 0.44^ab^	73.47 ± 0.29^a^
Crude protein	15.18 ± 0.38^a^	17.00 ± 0.44^b^	15.81 ± 0.28^ab^	15.85 ± 0.23^ab^	14.91 ± 0.11^a^
Crude lipid	4.56 ± 0.31	5.68 ± 0.33	4.60 ± 0.63	5.23 ± 0.21	4.81 ± 0.07
Ash	5.61 ± 0.36	5.72 ± 0.12	5.34 ± 0.34	5.61 ± 0.31	4.83 ± 0.27

*Note*: The values presented were average ± standard deviation (*n* = 4). Different superscript letters indicate significant differences at *P* < 0.05.

**Table 4:** Mucosal fold height and mucosal fold surface of tilapia after fed for 90 days with different dietary supplementation level of LP20.

	10 mg/kg	20 mg/kg	100 mg/kg	250 mg/kg	0 (control)
Mucosal fold height (*μ*m)	51 ± 5^a^	122 ± 12^b^	74 ± 7^a^	55 ± 3^a^	55 ± 6^a^
Mucosal fold surface area (*μ*m^2^)	1462 ± 204^a^	4839 ± 364^b^	1972 ± 233^a^	1627 ± 100^a^	1271 ± 102^a^

*Note*: The values presented were average ± standard deviation (*n* = 4). Different superscript letters indicate significant differences at *P* < 0.05.

**Table 5:** Red blood cell count (RBC), white blood cell count (WBC), hemoglobin concentration (Hb), and hematocrit value (Ht) of tilapia fed with diets supplemented with different levels of LP20 upon challenged *Streptococcus agalactiae*.

	RBC (×10^6^ cell/mL)	WBC (×10^4^ cell/mL)	Hb (g/dL)	Ht (%)
10 mg/kg	1.40 ± 0.16^a^	3.90 ± 0.36^ab^	7.80 ± 1.11	27.31 ± 8.32
20 mg/kg	1.27 ± 0.04^a^	3.45 ± 0.21^a^	6.00 ± 0.28	25.00 ± 1.31
100 mg/kg	2.00 ± 0.53^ab^	5.30 ± 0.56^b^	7.53 ± 0.31	28.60 ± 4.40
250 mg/kg	2.02 ± 0.31^ab^	5.20 ± 0.78^b^	6.60 ± 0.60	24.59 ± 3.36
Positive control	1.98 ± 0.34^ab^	3.20 ± 0.34^a^	6.60 ± 1.43	27.46 ± 3.69
Negative control	2.55 ± 0.34^b^	4.60 ± 0.62^b^	5.95 ± 0.82	28.48 ± 1.80

*Note*: The values presented were average ± standard deviation (*n* = 4). Different superscript letters indicate significant differences at *P* < 0.05.

## Data Availability

The data used to support the findings of this study are available from the corresponding author upon request.

## Supplementary Materials

Supplementary Materials Table S1: The range of water quality parameters (mean ± standard deviation) of tilapia fed with different supplementation levels of LP20.
