# Supplementary material for: Evaluation of Growth and Health Performance of Juvenile Tilapia Oreochromis sp. Fed with Various Supplementation of Heat-Killed Lactobacillus plantarum
Source: Aquac Nutr. 2023 Aug 22;2023:8860364. doi: 10.1155/2023/8860364 (PMC10465244; doi:10.1155/2023/8860364)
Supplement: Supplementary Materials — Table S1: The range of water quality parameters (mean ± standard deviation) of tilapia fed with different supplementation levels of LP20. [file 8860364.f1.pdf]

SUPPLEMENTARY MATERIAL

Supplementary table 1. The range of water quality parameters (mean ± standard deviation) of tilapia fed with different supplementation levels of LP20.

Treatment	Temperature (°C)	pH	Dissolved oxygen (mg/L)	Nitrite (mg/L)	Nitrate (mg/L)	TAN (mg/L)
10 mg/kg	27.50-29.90 (28.56±0.09)	7.83-8.30 (8.04±0.10)	6.18-8.40 (7.32±0.20)	0.22-3.17 (1.99±0.40)	< 10	0.65-0.85 (0.74±0.03)
20 mg/kg	27.43-30.40 (28.57±0.14)	7.80-8.30 (8.04±0.06)	6.05-8.35 (7.26±0.22)	0.18-2.84 (1.98±0.11)	< 10	0.76-0.84 (0.80±0.06)
100 mg/kg	27.18-30.38 (28.57±0.08)	7.83-8.30 (8.07±0.09)	6.30-8.40 (7.41±0.23)	0.09-3.47 (2.88±0.14)	< 10	0.70-0.82 (0.77±0.04)
250 mg/kg	27.08-30.58 (28.56±0.03)	7.75-8.25 (8.00±0.05)	6.30-8.28 (7.30±0.09)	0.13-3.40 (2.27±0.17)	< 10	0.78-0.84 (0.81±0.03)
0 (control)	26.88-29.35 (28.43±0.06)	7.70-8.23 (7.97±0.11)	5.98-8.33 (7.23±0.29)	0.15-3.28 (2.25±0.28)	< 10	0.73-0.87 (0.80±0.09)
